# Contribution of transcription factor, SP1, to the promotion of HB-EGF expression in defense mechanism against the treatment of irinotecan in ovarian clear cell carcinoma

**DOI:** 10.1002/cam4.301

**Published:** 2014-07-24

**Authors:** Kohei Miyata, Fusanori Yotsumoto, Sung Ouk Nam, Takashi Odawara, Sadao Manabe, Toyokazu Ishikawa, Hiroaki Itamochi, Junzo Kigawa, Shuji Takada, Hiroshi Asahara, Masahide Kuroki, Shingo Miyamoto

**Affiliations:** 1 Department of Obstetrics and Gynecology, Faculty of Medicine, Fukuoka UniversityFukuoka, Japan; 2 Department of Biochemistry, Faculty of Medicine, Fukuoka UniversityFukuoka, Japan; 3 Central Research Institute for Advanced Molecular Medicine, Fukuoka UniversityFukuoka, Japan; 4 Department of Systems BioMedicine, National Research Institute for Child Health and DevelopmentTokyo, Japan; 5 Kanonji Institute Research Foundation for Microbial Diseases of Osaka UniversityKagawa, Japan; 6 Department of Obstetrics and Gynecology, Tottori University School of MedicineTottori, Japan; 7 Department of Cancer Center, Tottori University HospitalTottori, Japan; 8 Department of Systems BioMedicine, Graduate School of Medical and Dental Sciences, Tokyo Medical and Dental UniversityTokyo, Japan

**Keywords:** Chemotherapy, gene transcriptional regulation, HB-EGF, ovarian cancer

## Abstract

Ovarian clear cell carcinoma (OCCC) is a worst histological subtype than other ovarian malignant tumor. Heparin-binding epidermal growth factor-like growth factor (HB-EGF) is a promising target for ovarian cancer therapy. The aims of this study were to validate the efficacy of HB-EGF–targeted therapy for OCCC and to identify the transcription factor that contributed to the induction of HB-EGF by SN38 treatment in OCCC cells. HB-EGF was highly expressed in OCCC cells, and an increase of HB-EGF was induced by SN38 which had only antitumor effect among conventional anticancer agents on OCCC. A specific inhibitor of HB-EGF, a cross-reacting material 197 (CRM197), led to a synergistic increase in the number of apoptotic OCCC cells with the treatment of SN38. The luciferase assay with 5′-deletion promoter constructs identified a GC-rich element between −125 and −178 (the distal transcription start site was denoted +1) as a *cis*-regulatory region, and the treatment of SN38 induced luciferase activity in this region. An in silico and chromatin immunoprecipitation analysis estimated that SP1 bound to the *cis*-regulatory region of *HB-EGF* in OCCC cells. Real-time PCR and cell viability assays showed that the transfection of a small interfering RNA targeting SP1 suppressed the expression of HB-EGF induced by SN38, resulting in the enhanced sensitivity of SN38. Taken together, these results indicate that induction of HB-EGF expression contributed to defense mechanism against treatment of SN38 through the transcriptional activity of SP1 in OCCC cells.

## Introduction

Ovarian cancer is the most lethal gynecological cancer in the Western world. Ovarian cancer usually has a poor prognosis because many cases are diagnosed in advanced stages. Standard treatment involves surgery, followed by chemotherapy including platinum derivatives (e.g., cisplatin [CDDP] or carboplatin) and taxanes (e.g., paclitaxel [PTX] or docetaxel). However, the 5-year survival rate of patients with advanced ovarian cancer has remained for the past three decades 1. Ovarian clear cell carcinoma (OCCC), which is a common histological type in Japanese, is known to indicate significant drug resistance for conventional anticancer agents, resulting in a poorer prognosis. Moreover, the occurrence of OCCC, which links to endometriosis, has rapidly increased throughout the world 2. Therefore, a new strategy for OCCC therapy has been highly anticipated 2.

Heparin-binding epidermal growth factor-like growth factor (HB-EGF), which belongs to the EGF family, is synthesized as an 87 amino acid membrane-anchored glycoprotein 3–5. Cleavage of membrane-anchored HB-EGF by proteases, called “ectodomain shedding,” induces the secretion of soluble mature form of HB-EGF from the cell surface. Through ectodomain shedding, HB-EGF contributes to several biological processes, such as wound healing 6, inflammation 7,8, embryogenesis 9, and carcinogenesis 10. In previous reports, we described that the expression of HB-EGF was higher than that of other EGFR ligands in the ascites and tissues of ovarian cancer patients 11,12. However, the molecular mechanism(s) underlying the increase of HB-EGF expression in ovarian cancer has remained unclear.

There have been several reports that have described other molecular mechanisms affecting the expression of HB-EGF. For example, inflammatory cytokines, including tumor necrosis factor-*α* and interleukin-1*β*, directly or indirectly activated the transcription of HB-EGF 13,14. In bladder cancer cells, insulin induces the transcriptional activation of epiregulin, HB-EGF, and amphiregulin (AREG), which may be regulated by transcriptional factors including specific protein 1 (SP1), activator protein-1 (AP-1), and nuclear factor-kappa B (NF-*κ*B) 15. HB-EGF was rapidly induced following exposure to a variety of chemotherapeutic agents, and the chemotherapy-induced HB-EGF expression was largely dependent on AP-1 and NF-*κ*B 16. In addition, transcription factors including SP1, AP-1, pancreatic and duodenal homeobox-1 (PDX-1), and myogenic differentiation 1 (MyoD) were detected as direct regulators of HB-EGF expression 15,17–19. However, the molecule(s) directly involved in the transcript of HB-EGF has remained unknown in human cancer.

The aim of this study is to assess the association between HB-EGF expression and the cell viability of OCCC cells. Additionally, in order to elucidate transcriptional regulation of HB-EGF in OCCC cells, we also performed a screening assay and identified the transcription factor, SP1, which is responsible for the upregulation of HB-EGF induced by anticancer agent.

## Materials and Methods

### Cells and cell culture

Eleven OCCC cell lines (RMG-I, RMG-II, OVISE, OVTOKO, KK, TU-OC-1, KOC-7C, HAC-2, OVAS, SMOV-2, ES-2) and 293T cells were used for the studies. The KK cells were a kind gift from Professor Yoshihiro Kikuchi, National Defense Medical College, Japan; the OVAS cells were provided by Professor Hiroshi Minaguchi, Yokohama City University, Japan; KOC-7C by Dr. Toru Sugiyama, Kurume University, Japan; SMOV-2 by Dr. Tomohiro Iida, St. Marianna University, Japan; and HAC-2 by Dr. Masato Nishida, Tsukuba University, Japan. TU-OC-1 cells were derived as described previously 20. ES-2 cells were purchased from Summit Pharmaceuticals International Corporation (Tokyo, Japan). RMG-I, RMG-II, OVISE, OVTOKO, and 293T cells were purchased from the Health Science Research Resources Bank (HSRRB, Osaka, Japan). All OCCC cell lines were maintained in RPMI-1640 medium (Gibco, Carlsbad, CA), and 293T cells were maintained in DMEM medium (Gibco). The culture media were supplemented with 10% fetal bovine serum (FBS) (ICN Biomedical, Irvine, CA).

### Cell viability and apoptosis assay

Paclitaxel (PTX), cisplatin (CDDP), and SN38 (the active metabolite of irinotecan) were purchased from Sigma-Aldrich (St. Louis, MO). Recombinant human HB-EGF (rhHB-EGF) was purchased from R&D systems (Minneapolis, MN). Cross-reactive material 197 (CRM197), a specific inhibitor of HB-EGF, was prepared as described previously 21. In the cell viability assay, 20,000–40,000 cells were incubated in RPMI-1640 medium with 5% FBS and different concentrations of PTX, CDDP, or SN38 on 12-well plates. After a 72-h incubation, the cells were counted using a hemocytometer after the addition of trypan blue exclusion dye to determine the cell viability. In the apoptosis assay, TUNEL-positive cells were quantified as apoptotic cells by a flow cytometric analysis as described previously 22.

### DNA extraction, RNA isolation, and cDNA synthesis

ES-2 cells were suspended in 0.1% SDS/Tris-EDTA buffer (pH 8.0). After treatment with proteinase K (Sigma-Aldrich) and RNase A (Invitrogen, Carlsbad, CA), phenol/chloroform extraction and ethanol precipitation were preformed to purify genomic DNA. Total RNA was isolated using TRIzol reagent (Invitrogen) according to the manufacturer's protocol. First-strand cDNA synthesis was performed with 1 *μ*g of total RNA using with Primescript II reverse transcriptase (Takara Bio, Otsu, Shiga, Japan) following the manufacturer's protocol.

### Real-time quantitative PCR (qPCR)

qPCR was performed using TaqMan Universal PCR Master Mix (Applied Biosystems, Foster City, CA) and the samples were analyzed by an Applied Biosystems 7500 Fast Real-time PCR system. TaqMan probes used were as follow: *HB-EGF*: Hs00181813_m1; *AREG*: Hs00950 669_m1; and *GAPDH*: Hs02758991_g1. The methods used for the data analysis were described previously 12.

### Western blot analysis

Whole cell lysates (5 *μ*g) were electrophoresed through 10% SDS-PAGE gels and proteins were transferred to a polyvinylidene difluoride membrane using a semi-dry electroblot (Bio-Rad, Hercules, CA). The blot was probed with primary antibodies (anti-human SP1 antibody [#07-645; Millipore, Billerica, MA], anti-human GAPDH antibody [#ABS16; Millipore]) and secondary antibody (anti-rabbit IgG-Peroxidase [A9169; Sigma-Aldrich]), and then the binding to antibody was detected using ECL Western Blotting Detection Regents (General Electric Company, Fairfield, CT) and a Fuji image analyzer (LAS mini-3000; Fujifilm, Tokyo, Japan) according to the manufacturer's protocol.

### ELISA

Cells were incubated with culture medium for 24 h. After a 24-h incubation, the concentrations of HB-EGF and AREG in the culture medium were determined using a commercially available sandwich ELISA (DuoSet kit; R&D Systems) according to the manufacturer's instructions.

### Vectors

To generate promoter constructs for the *HB-EGF* gene promoter, which were located at −4138 to +205 base pair (bp), −125 to +205 bp, −178 to +205 bp, and −253 to +205 bp from its transcriptional start site (TSS), the sequences were amplified and cloned into pGL4.12 (Promega, Madison, WI). All nucleotide numbering was done with reference to the TSS. The primers used for these PCR assays are listed in Table S1. The pGL4.12 and fragments were digested with *Kpn*I and *Nco*I restriction enzymes and ligated. To create pGL/HB_−2585/+205_ and pGL/HB_−454/+205_, pGL/HB_−4138/+205_ was digested with *Nde*I and *Kpn*I or *Ase*I and *Kpn*I, and self-ligation was performed after blunt-ended treatment with Klenow fragment (Takara Bio). Site-detected mutants of the reporter vector were constructed by inverse PCR with primers described in Table S1. The sequences of all generated vectors were confirmed by DNA sequencing. The expression vectors for SP1 (EX-Y2188-Lv105) and EGFP (EX-EGFP-Lv105) were purchased from GeneCopoeia (Rockville, MD).

### Reporter assay

The luciferase assay was performed with ES-2 cells and 293T cells. A total of 25,000 cells were incubated on 24-well plates for 16 h, and then were cotransfected with 1 *μ*g of promoter reporter vector and 25 ng of pRL-TK (Promega), which had a renilla-luciferase gene downstream of the thymidine kinase promoter. Lipofectamine 2000 (Invitrogen) and Opti-MEM (Gibco) were used for transfection. When cells were treated with SN38, the culture medium was changed to RPMI-1640 medium supplemented with 10% FBS and SN38 (10 nmol/L). After a 48-h incubation, Dual-Luciferase Reporter Assay (Promega) was performed according to the manufacturer's protocol.

### Small interfering RNA

Stealth RNAi (Invitrogen) against SP1 (siSP1_1: 5′-CAAUGGCAGUGAGUCUUCCAAGAAU-3′, siSP1_2: 5′-GACAGGUCAGUUGGCAGACUCUACA-3′) and control siRNAs (Control_1: Stealth RNAi siRNA Negative Control Lo GC, Control_2: Stealth RNAi siRNA Negative Control Med GC, Invitrogen) were transfected using Lipofectamine RNAiMAX (Invitrogen) according to the manufacturer's protocol.

### Chromatin immunoprecipitation assay

ES-2 cells which were cultured until subconfluent in five 15 cm dishes were fixed in 1% formaldehyde for 10 min at 37°C. Chromatin was sheared using a Digital Sonifier S-250D (Branson, Danbury, CT) to yield DNA fragments of 300–800 bp. Nuclear extraction, immunoprecipitation, reverse cross-linking, and DNA purification were performed using Magna ChIP A kit (Millipore) according to the manufacturer's protocol. An anti-SP1 antibody (#07-645; Millipore), tri-methyl-histone H3 (Lys4) monoclonal antibody (#9751S; Cell Signaling Technology, Boston, MA), and normal rabbit IgG (#2729S; Cell Signaling Technology) were used in the immunoprecipitation reactions. Quantitative real-time PCR was performed using SYBR Premix Ex Taq GC (Takara Bio) to detect the HB-EGF promoter fragments.

### Statistical analysis

The two-tailed independent Student's *t*-test was used to calculate all *P*-values. *P* < 0.05 was considered statistically significant.

## Results

### Promotion of HB-EGF expression in response to SN38 treatment

**Figure 1:**
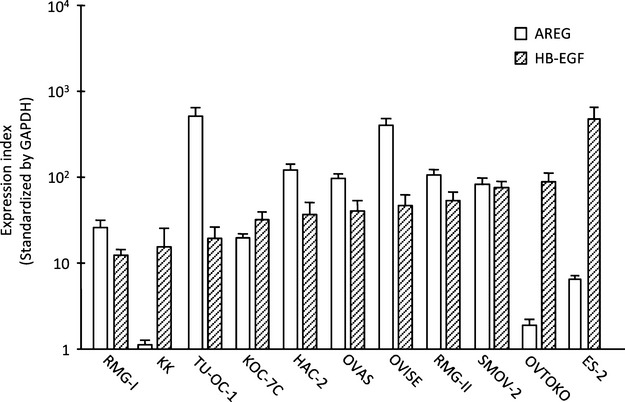
The expression of HB-EGF in 11 ovarian clear cell carcinoma (OCCC) cell lines. The real-time PCR data show the expression of heparin-binding epidermal growth factor-like growth factor (HB-EGF) and amphiregulin (AREG) in OCCC cells. Each value represents the mean (*n* = 3) and standard deviation (SD) of the mRNA expression index for HB-EGF (diagonal striped bars) and AREG (open bars).

First, we examined the expression of HB-EGF and AREG in 11 cell lines of OCCC. HB-EGF was highly expressed in all of the cell lines, and eight of the 11 cell lines had a high-expression level of AREG (Fig.1). OVTOKO and ES-2 cells had the highest expression of HB-EGF, while the OVISE and RMG-II cells had higher expression of AREG compared to that of HB-EGF.

**Figure 2:**
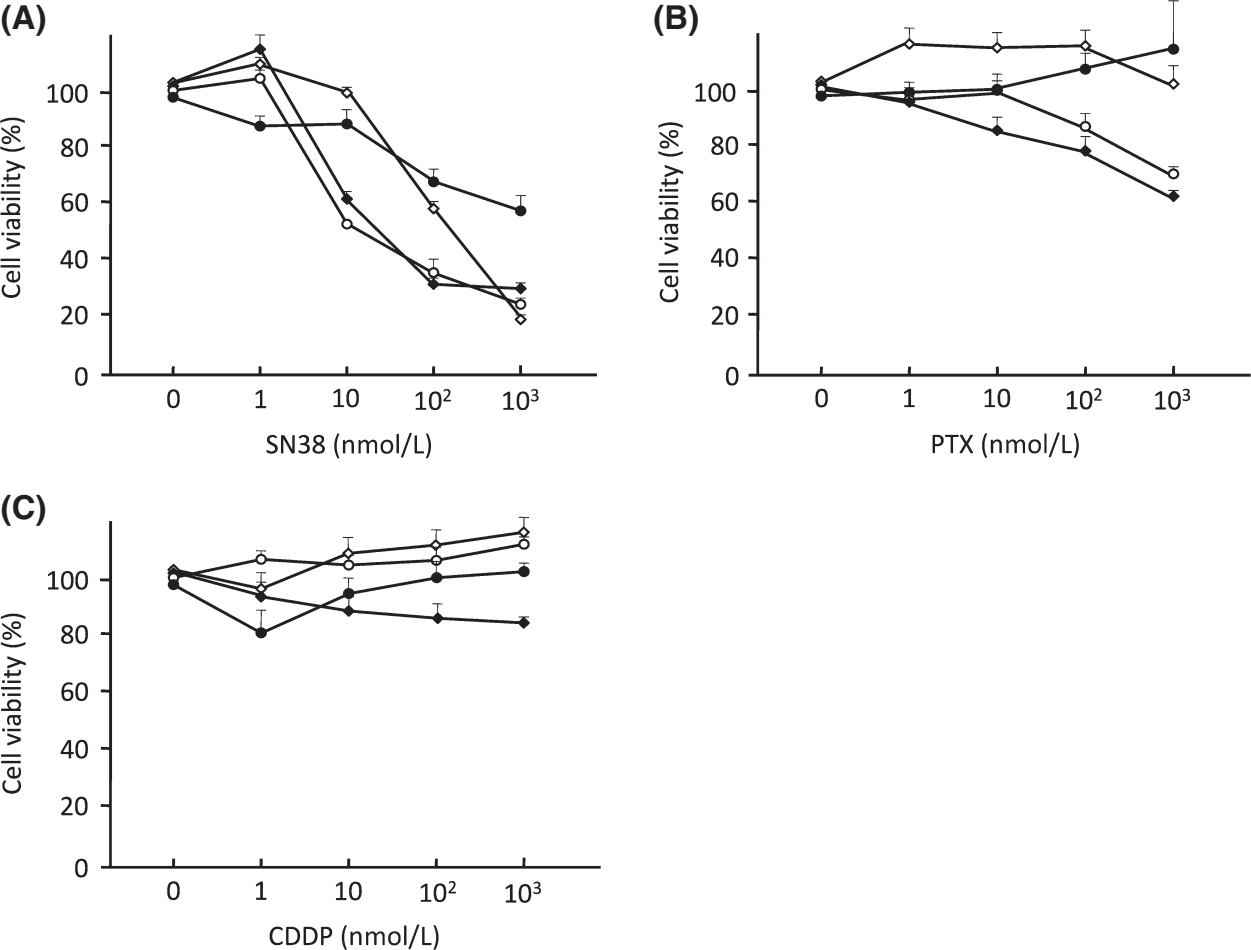
The efficacy of conventional anticancer agents against OCCC cells. Differences in the viability of OVISE (closed squares), RMG-II (closed circles), OVTOKO (open squares), and ES-2 (open circles) OCCC cells after treatment with SN38 (A), paclitaxel (PTX; B), and cisplatin (CDDP; C) for 72 h. Each value represents the mean cell viability rate (*n* = 3) and the SD. OCCC, ovarian clear cell carcinoma.

**Figure 3:**
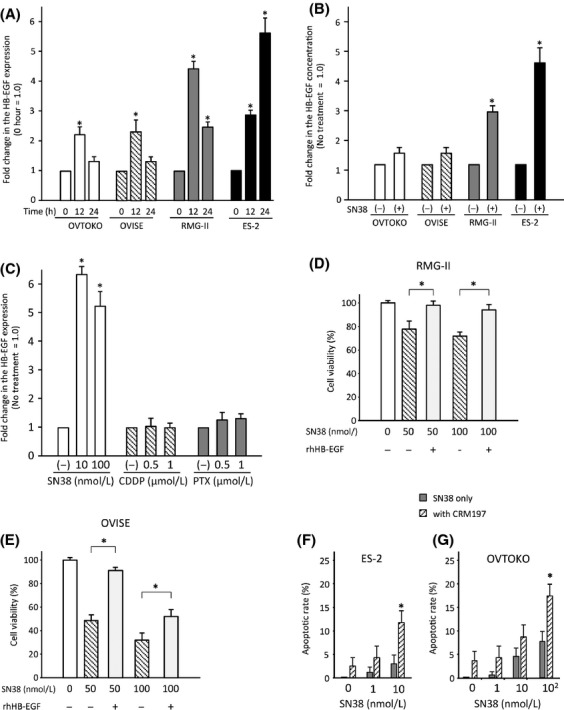
The association between HB-EGF expression and the SN38 treatment of OCCC cells. The induction of HB-EGF mRNA in cells (A) and HB-EGF protein in the culture medium (B) in OVTOKO (open bars), OVISE (diagonal striped bars), RMG-II (gray bars), and ES-2 (closed bars) cells treated with SN38 (10 nmol/L) for 12 or 24 h. Each value represents the mean (*n* = 3) and SD. (C) The differences in the HB-EGF mRNA expression in ES-2 cells after the treatment with SN38 (open bars), CDDP (diagonal striped bars), or PTX (gray bars). The differences in cell viabilities of RMG-II (D) and OVISE (E), which were cultured with recombinant human HB-EGF (0 and 200 pg/mL) and SN38 (0, 50, and 100 nmol/L). Each value represents the mean fold change (*n* = 3) and the SD. *P < 0.05. The changes in the apoptosis rate of ES-2 (F) and OVTOKO (G) cells after the treatment with each concentration of SN38 with (closed bars) or without CRM197 (10 *μ*g/mL, diagonal striped bars). Each value represents the mean apoptosis ratio (*n* = 3) and SD. *P < 0.05, SN38-only versus SN38 with CRM197. HB-EGF, heparin-binding epidermal growth factor-like growth factor; PTX, paclitaxel.

To evaluate in vitro anticancer effects of conventional anticancer agents in the OVISE, RMG-II, OVTOKO, and ES-2 cells, cell viability assays were performed using SN38 (Fig.2A), PTX (Fig.2B), or CDDP (Fig.2C). In this analysis, SN38 was a most effective anticancer agent in all four OCCC cell lines. Real-time PCR showed a twofold or higher increase in HB-EGF expression induced by the treatment of the OCCC cells with SN38, and the concentration of HB-EGF also increased more than twofold in the culture medium of RMG-II and ES-2 cells following SN38 treatment (Fig.3A and B). In contrast, a high concentration of PTX or CDDP did not induce HB-EGF expression in ES-2 cells (Fig.3C). The addition of the recombinant HB-EGF in cell culture blocked a decrease in cell viability with the treatment of SN38 in OCCC cells (Fig.3D and E). These results indicated that HB-EGF plays a pivotal role in defense mechanism against the treatment of SN38 in OCCC cells.

To address the potential synergistic anticancer effects of the combination of SN38 and a specific inhibitor of HB-EGF (CRM197), apoptosis assays were performed after treating ES-2 or OVTOKO cells with SN38 and/or CRM197. Treatment with 10 *μ*g/mL of CRM197 and 10 nmol/L of SN38 induced a marked increase in the number of apoptotic ES-2 and OVTOKO cells, compared to cells with SN38 treatment alone, as determined using TUNEL assay (Fig.3F and G). These results suggested that the suppression of HB-EGF during the treatment with SN38 leads to a synergistic anticancer effect in OCCC cells.

### Screening for transcription factors that regulate the HB-EGF expression induced by SN38 treatment

**Figure 4:**
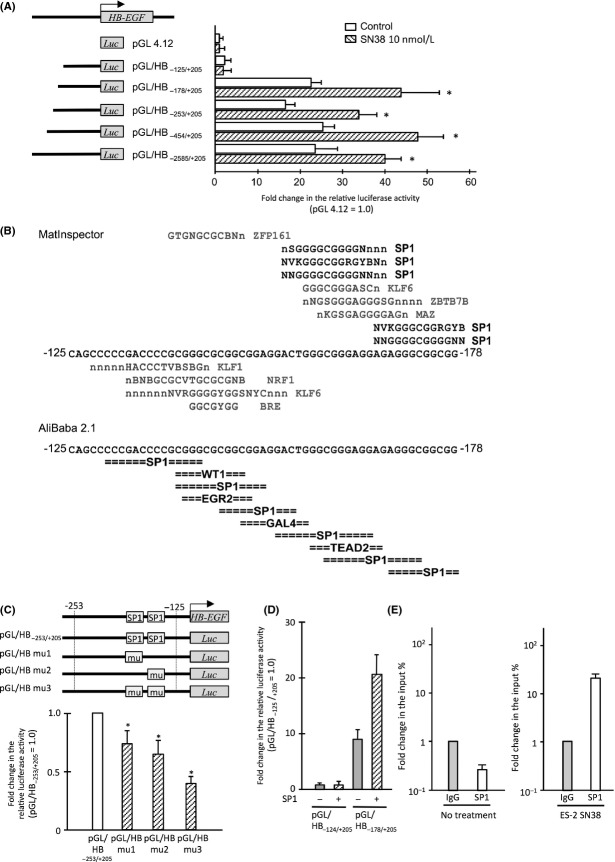
The interaction of SP1 with the *HB-EGF* promoter region following SN38 treatment. (A) The alterations in the luminescence of ES-2 cells for each reporter vector in cells with (open bars) and without (diagonal striped bars) SN38 (10 nmol/L) treatment. A schematic diagram showing the reporter vectors on the left side. (B) A schematic diagram obtained from the in silico analysis including the MatInspector and AliBaba 2.1 programs. (C) The luciferase reporter analysis of the site mutants in ES-2 cells. The schematic diagram shows the wild-type SP1-binding sites as “SP1” and the mutated binding sites as “mu” in each vector. The alterations in the luminescence of ES-2 cells among the wild-type reporter vector (open bar) and the mutated reporter vectors (diagonal striped bars) in the presence of SN38 (10 nmol/L). Each value represents the mean (*n* = 3) and SD. (D) The differences in the relative luciferase activity of 293T cells transfected with pGL/HB_−178/+205_ and the SP1 expression vector (diagonal striped bars). An EGFP expression vector was used as a control (gray bars). Each value represents the mean (*n* = 3) and SD. All relative luciferase activities were normalized to the renilla luminescence. (E) The results of the ChIP analysis of ES-2 cells with or without SN38 (10 nmol/L) treatment. The fold change in the immunoprecipitated DNA for SP1 (open bars) against templates due to the nonspecific binding with IgG (gray bars). Each value represents the mean (*n* = 3) and SD. *P < 0.05, versus control. HB-EGF, heparin-binding epidermal growth factor-like growth factor; SP1, specific protein 1.

To identify transcription factor(s) that contribute to the HB-EGF induction following treatment with SN38, a promoter analysis was performed using the ES-2 cells. For the reporter gene analysis, a *HB-EGF* promoter fragment (−2585/+205), which is conserved among mammalian species, fused to a luciferase vector, and various truncated constructs were synthesized. The luciferase assay showed that a reporter vector containing promoter fragment of −178/+205 bp from HB-EGF TSS (pGL/HB_−178/+205_) exhibited an about 20-fold increase in luciferase activity compared to that of pGL/HB_−125/+205_ (Fig.4A). Additionally, treatment with SN38 induced ∼twofold increase in the luciferase activity in a reporter vector containing pGL/HB_−178/+205_ compared to the control (Fig.4A). Accordingly, the sequence located −178 to −125 bp from the TSS of *HB-EGF* was recognized as a promoter sequence bound by transcription factors.

Using the MatInspector (http://www.genomatix.de) and AliBaba 2.1 (http://www.gene-regulation.com) in silico analysis programs, we found that SP1-binding sites are commonly found in this promoter sequence (Fig.4B). Since the HB-EGF promoter has a TATA-less and GC-rich promoter, which are characteristics of SP1-binding promoters, we focused on SP1 as the candidate transcription factor inducing HB-EGF in SN38-treated ES-2 cells 17,23,24.

Reporter vectors with site mutations of each SP1-binding site showed significantly reduced luciferase activity compared to the control (Fig.4C). Moreover, the luciferase activity significantly decreased, compared to the control, when the two putative binding sites of SP1 were replaced with mutant sites (Fig.4C). Forced expression of SP1 activated the luciferase activity in 298T cells transfected with pGL/HB_−178/+205_, although no difference was found between the forced expression of SP1 and control in 293T cells transfected by pGL/HB_−125/+205_ (Fig.4D).

To detect direct interaction between the SP1 protein and HB-EGF promoter in ES-2 cells, a chromatin immunoprecipitation assay was performed in cells with or without treatment with SN38. Quantitative real-time PCR showed that, during treatment with SN38, the level of HB-EGF promoter bound to SP1 significantly increased compared to that of control cells (Fig.4E). These results indicated that the HB-EGF expression promoted by the treatment with SN38 was regulated by SP1 in OCCC cells.

### Contribution of SP1 to the HB-EGF expression and SN38 sensitivity of OCCC cells

**Figure 5:**
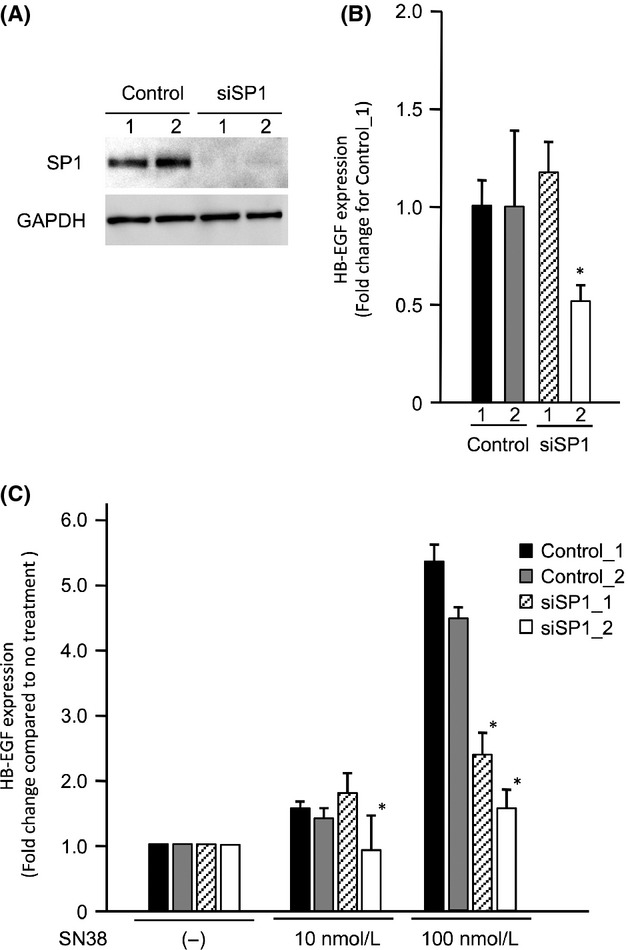
The contribution of SP1 to the HB-EGF induction following the treatment with SN38. (A) A Western blot analysis was performed to confirm the SP1 reduction in the transfected ES-2 cells induced by treatment with siRNAs against SP1 or a control siRNA. (B) The alterations in the HB-EGF mRNA expression in the transfected ES-2 cells induced by treatment with the siRNAs against SP1 or the control siRNA. (C) The induction of HB-EGF mRNA in the RMG-II cells transfected with siRNAs against SP1 or the control siRNA following the treatment of SN38. Each value represents the mean (*n* = 3) and SD. Closed bars, control_1 siRNA; gray bars, control_2 siRNA; diagonal striped bars, siSP1_1 si RNA; open bars, siSP1_2 siRNA. *P < 0.05, control_1 versus each siRNA. SP1, specific protein 1.

**Figure 6:**
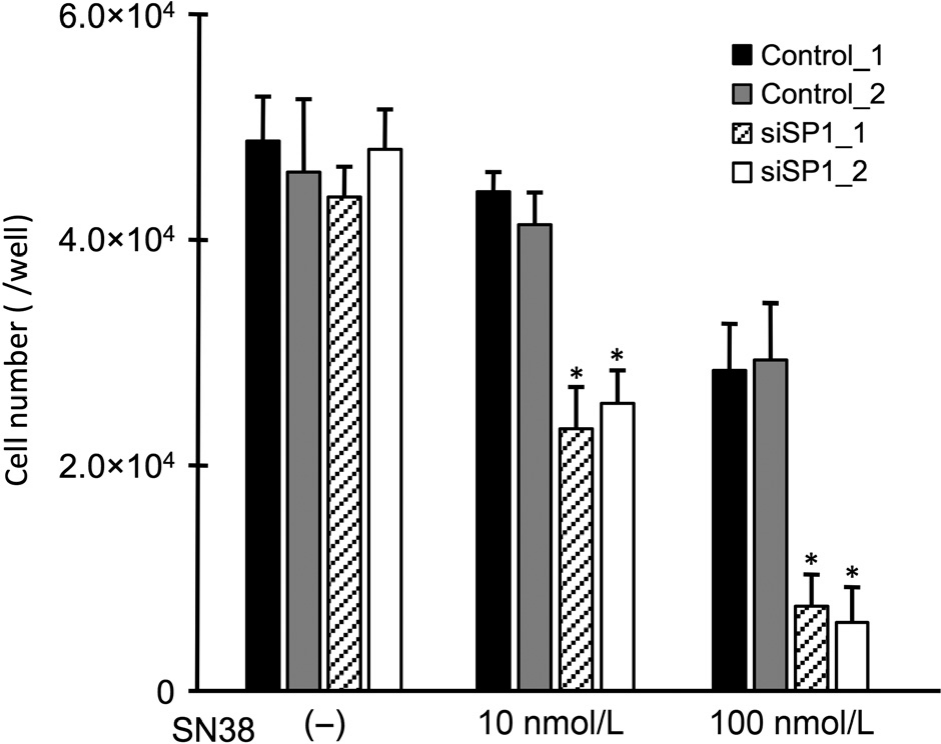
The contribution of SP1 to the cell viabilities following the treatment with SN38. The alterations in the numbers of RMG-II cells transfected with siRNAs against SP1 or a control siRNA after treatment with SN38. Each value represents the mean (*n* = 3) and SD. Closed bars, control_1 siRNA; gray bars, control_2 siRNA; diagonal striped bars, siSP1_1 si RNA; open bars, siSP1_2 siRNA. *P < 0.05, control_1 versus each siRNA. SP1, specific protein 1.

To confirm whether SP1 affects the sensitivity of cells to SN38 via the induction of HB-EGF expression, the expression and the cell viability assays were performed after the transfection of small interfering RNA (siRNA) targeting SP1. A Western blot analysis showed that the ES-2 cells transfected with siSP1_1 and siSP1_2 had reduced expression of the SP1 protein compared to the cells transfected with control siRNAs (Fig.5A). In the ES-2 cells transfected with siSP1_2, the HB-EGF expression was reduced to approximately half the control level, although the HB-EGF expression was not altered in the ES-2 cells transfected with siSP1_1 (Fig.5B). No induction of HB-EGF expression was found in the presence of SN38 in the ES-2 cells transfected with either siSP1_1 or siSP1_2 (Fig.5C). Additionally, we examined alterations in the cell viabilities using RMG-II cells, which had the least antitumor effect with the treatment of SN38. Following the treatment with SN38, the number of RMG-II cells transfected with siSP1_1 and siSP1_2 significantly decreased compared to that in the RMG-II cells transfected with control siRNAs (Fig.6). These results indicate that SP1 regulates the drug sensitivity of SN38 by regulating the HB-EGF expression in OCCC cells.

## Discussion

In this study, HB-EGF was attributable for the escape from cell death, as SN38 damaged OCCC cells. SP1 activated HB-EGF expression through its binding to multiple transcription sites within the promoter of HB-EGF in OCCC cells. In addition, the suppression of HB-EGF as well as SP1 enhanced the sensitivity of SN38 in OCCC cells.

SP1, which belongs to the family of SP1/Kruppel-like factor (KFL) transcriptional factors, regulates the expression of numerous genes involved in cell proliferation, apoptosis, and differentiation 25. Phorbol 12-myristate 13 acetate (PMA) induced an increase in the transcriptional activity of SP1 through the deacetylation of SP1, and also provoked the ectodomain shedding of HB-EGF; it also increased the expression of HB-EGF 26,27. Additionally, NF-*κ*B induced the expression of HB-EGF, and the NF-*κ*B and SP1 binding sequence was shown to be the same GC-rich element in colon cancer cells 16,28. This evidence suggests that SP1 augments the expression of HB-EGF through the deacetylation of SP1 or via an interaction with NF-*κ*B. In mice, SP1 was reported to be directly bound to the promoter regions of HB-EGF 24. In humans, SP1 also functions as a direct regulator for the expression of HB-EGF, possibly through various posttranslational modifications of SP1 and by interactions with other transcriptional factors.

Dysregulation of SP1 is found in many types of cancer, including ovarian, breast, and gastric cancer, for which HB-EGF is a rational therapeutic target 22,29,30. The hypoxia-inducible factor 2*α* (HIF2*α*)–SP1 complex activated coagulation factor VII promoter in OCCC and estrogen receptor *α* also form complexes with SP1 in other types of ovarian cancer 31,32. Additionally, previous reports showed that activation of SP1 promoted breast cancer development and the metastasis of gastric cancer 33,34. On the other hand, the SP1 level was highly upregulated in patients with early stage and minimally invasive lung cancer cells and in patients with stage I lung cancer compared to that in lung cancers with high invasiveness and in patients with stage IV lung cancer 35. These previous findings show that the significance of SP1 involvement in cancer progression have been controversial. In this study, the suppression of SP1 enhanced the sensitivity of OCCC cells to SN38. Therefore, it seems to be important that the suppression of SP1 itself, as well as the inhibition of the posttranslational modifications of SP1, are critical for cancer therapy.

Several compounds that inhibit the transcriptional activity of SP1 have been developed for cancer therapy 25, for example, arsenic trioxide downregulates the expression of SP1 36. A phase III trial of arsenic trioxide was performed for patients with acute promyelocytic leukemia classified as having low-to-intermediate risk, and the results suggested that all-*trans*-retinoic acid plus arsenic trioxide may be superior to all-*trans*-retinoic acid plus other chemotherapy 37. Bortezomib has been already used for the treatment of patients with multiple myeloma 38,39. Bortezomib has been shown to decrease the expression of SP1 and disrupt the interaction of SP1 with NF-*κ*B 40. Other inhibitory compounds for SP1 will be clinically applied for various diseases, in addition to cancer 41,42.

We have performed a clinical phase II trial using CRM197 in patients with recurrent and advanced ovarian cancer. To explore the importance of the posttranslational modifications of SP1 in the induction of HB-EGF expression and to search for compounds that can inhibit such modifications, a preclinical study should be performed using combination therapy with CRM197, irinotecan, and a compound that inhibits SP1, such as arsenic trioxide or bortezomib. Such a combination would likely improve the prognosis of patients with OCCC.

## Supporting Information

**Table S1.** Primer sequences.
