# Supplementary material for: Contribution of transcription factor, SP1, to the promotion of HB-EGF expression in defense mechanism against the treatment of irinotecan in ovarian clear cell carcinoma
Source: Cancer Med. 2014 Jul 24;3(5):1159–69. doi: 10.1002/cam4.301 (PMC4302667; doi:10.1002/cam4.301)
Supplement: Supplementary file 1 — Table S1. Primer sequences. [file cam40003-1159-SD1.pdf]

Table S1. Primer sequences

Primer	Sequence
forward primer for the HB-EGF reporter vector	GCCCATGGTCCCGCACCGAGAGG
reverse primer for pGL/HB$_{-4138/+205}$	GCGGTACCAATGAGAAGGCAGCTGAA
reverse primer for pGL/HB$_{-125/+205}$	GCGGTACCGCTGCCGGCGCCGCGAGCCG
reverse primer for pGL/HB$_{-178/+205}$	GCGGTACCCGCCGCCCTCTCCTCCC
reverse primer for pGL/HB$_{-253/+205}$	GCGGTACCTCCGCCACCTGCCGGTC
forward primer for pGL/HB mu1	aataGCCCGCGGGGTCGGGGGCTG
reverse primer for pGL/HB mu1	CGGAGGACTGGGCGGGAGGAGAGGG
forward primer for pGL/HB mu2	ataCCGCGGGGTCGGGGGCTG
reverse primer for pGL/HB mu2	CGGCGGAGGACTGGGCGGGAGGAGA
forward primer for ChIP PCR	TCCCGTGCTGGGAAGCTCGC
reverse primer for ChIP PCR	TGCCTCGGCCTGGTCCCAAAA
